# Auditory Cue Based on the Golden Ratio Can Improve Gait Patterns in People with Parkinson’s Disease

**DOI:** 10.3390/s21030911

**Published:** 2021-01-29

**Authors:** Valeria Belluscio, Marco Iosa, Giuseppe Vannozzi, Stefano Paravati, Antonella Peppe

**Affiliations:** 1 Department of Movement, Human and Health Sciences, University of Rome “Foro Italico”, P.zza Lauro de Bosis 15, 00135 Rome, Italy; valeria.belluscio@gmail.com; 2 IRCSS Fondazione Santa Lucia, Via Ardeatina 306, 00179 Rome, Italystefano.paravati@gmail.com (S.P.); a.peppe@hsantalucia.it (A.P.); 3 Department of Psychology, Sapienza University of Rome, via dei Marsi 78, 00185 Rome, Italy

**Keywords:** gait analysis, locomotion, walking, external rhythm, fractal, physiological rhythm, internal rhythm, motor adaptation

## Abstract

The harmonic structure of walking relies on an irrational number called the golden ratio (ϕ): in healthy subjects, it coincides with the stride-to-stance ratio, and it is associated with a smooth gait modality. This smoothness is lost in people with Parkinson’s disease (PD), due to deficiencies in the execution of movements. However, external auditory cues seem to facilitate movement, by enabling the timing of muscle activation, and helping in initiating and modulating motor output. Based on a harmonic fractal structure of gait, can the administration of an auditory cue based on individual’s ϕ-rhythm improve, in acute, gait patterns in people with PD? A total of 20 participants (16 males, age 70.9 ± 8.4 years, Hoehn and Yahr stage-II) were assessed through stereophotogrammetry: gait spatio-temporal parameters, and stride-to-stance ratio were computed before, during, and after the ϕ-rhythm administration. Results show improvements in terms of stride length (*p* = 0.018), walking speed (*p* = 0.014), and toe clearance (*p* = 0.013) when comparing gait patterns before and after the stimulus. Furthermore, the stride-to-stance ratio seems to correlate with almost all spatio-temporal parameters, but it shows the main changes in the before–during rhythm comparison. In conclusion, ϕ-rhythm seems an effective cue able to compensate for defective internal rhythm of the basal ganglia in PD.

## 1. Introduction

Parkinson’s disease (PD) is a chronic, neurologic disorder which primarily results from the death of dopaminergic neurons in the substantia nigra pars compacta, leading to dopamine deficiency. This deficiency is responsible for the major PD motor symptoms [1], which usually appear as tremor at rest, akinesia or bradykinesia, rigidity, and postural instability [2]. These features are known to affect gait of people with PD, which is usually characterized by decreased foot clearance, reduced stride length and gait velocity, increased stance phase, and greater forward inclination of the trunk, compared to age-matched controls [3,4]. 

In healthy subjects, gait is mostly achieved by automatic processes, mainly controlled by the basal ganglia-brainstem-spinal cord stepping generator loop. The steady-state of gait, in particular, was shown to be consistent with the so-called intrinsic “kinetic melody” [5], a term which describes the idea that individual motor impulses are synthesized and combined into integral kinesthetic structures. In human body, harmonic properties of locomotor patterns facilitate the gait rhythm control, that can be activated and regulated by serotonergic neurons projections of the brain stem [6]. 

In people with PD, however, an alteration of this “melodic” flow of movement was observed [7,8], and the related irregular timing of walking pace suggested a disturbance of coordinated rhythmic locomotion [9]. This alteration is mainly the consequence of neurodegenerative processes that cause damages to several neuronal circuits, especially in the dopaminergic neurons of the basal ganglia and the spinal central pattern generators [10], modifying the smooth, automatized performance of complex operations. Basal ganglia, in fact, play significant roles in the production and control of automatic and well-learned movements: first of all, they generate internal cues fundamental for the preparation and maintenance of motor plans carried out without attention; second, they contribute to the preparation and maintenance of motor schemes [11]. Therefore, dopaminergic depletion and disruption of sensorimotor integration systems lead to deficiencies in the execution of movements: as a consequence, people with PD may become dependent on external stimuli to initiate and modulate the motor output [11]. This assumption is supported by evidences of how soliciting brain mechanisms that control timing and coordination of movements can actively facilitate the recovery of movement in patients with PD [12,13]. That is the case of external auditory stimuli (cues), that seem to facilitate movement by enabling the timing of muscle activation to synchronize to the temporal structure of rhythm, reducing the time required for the muscle to respond to a given motor command [8]. Therefore, this type of externally provided cue may be used as a replacement to the lost ‘internal clock’ to facilitate synchrony of movements [8]. 

This capacity of the auditory system to enhance motor performance, by inducing motor–sensory feedback signals that are able to recalibrate internal pacing, has been proved by several studies: in the presence of external regulatory rhythmical stimuli, in fact, people with PD show improvements in terms of cadence, walking speed, and stride length [12,14,15,16,17,18,19,20], therefore reducing gait deficits and risk of falls. 

Auditory cues are usually presented with a fixed-time design (i.e., metronome, rhythmic auditory stimulation), where the user has to get in phase with the cue [15]. It is therefore fundamental that the rhythm is effectively designed otherwise, if it is not well tuned to patients’ pace and inner timing, it would lose its therapeutic value [8]. In this respect, a recent study highlighted that, despite similar walking speed between healthy and PD participants, stance and double support phases increased with respect to the swing phase, impairing the ratio between the entire gait cycle and the stance phase, that in healthy gait was shown to converge to the so-called Golden Ratio [7], an irrational number close to 1.618034, often indicated with the Greek letter Phi (ϕ). The Golden Ratio ϕ, in healthy subjects, coincides with the proportion between an entire gait cycle and the stance phase (when the foot is in contact with the ground) [21] and highlights the smooth, “melodic” flow of movement. Alterations and loss of symmetry in this stride-to-stance ratio in people with PD were linked to a deficient internal cue production and inadequate contribution of the basal ganglia [7,12].

These characteristics related to gait performance have been commonly investigated using foot switch systems [3,14,16], but in the majority of works gait analysis was used through the adoption of optical systems [4,7,11,17,21], depth sensors [22] or GaitRite [23]. Gait analysis allows to detect even the smallest changes in gait patterns, such as in these works where it was used for comparing spatio-temporal parameters before and after an auditory cue administration. 

In this work we hypothesize that the adoption of a ϕ -based external auditory cue could provide additional benefits toward a restored harmonic structure of walking with respect to common binary auditory cues based on symmetry between strides. Gait phases, in fact, have been found in a strict relationship with harmonic properties of walking, demonstrating that the repetitive gait phases are in repetitive proportions with each other, facilitating the control of locomotion [21]. Therefore, the aim of the study was to verify through a gait analysis if the administration of an ad-hoc auditory stimulus, based on a personalized physiological intrinsic stride-to-stance rhythm, could improve gait patterns in people with PD. 

The hypothesis is that the stride-to-stance rhythm improves gait patterns in people with PD, and these improvements will last in time also immediately after the cue removal. 

## 2. Materials and Methods

The present study has been approved by the IRCSS Santa Lucia Ethical Committee (Protocol Number CE/PROG.678) and registered on ClinicalTrial.gov. Each participant gave his or her written informed consent for participating in the study. 

### 2.1. Population

A total of 20 people with a diagnosis of PD (16 males, 70.9 ± 8.4 years, Hoehn and Yahr stage II) were enrolled in the study. This sample size complied with the minimum number of participants recommended by a power analysis purposely performed (α = 0.05; power (1 − β) = 0.95, effect size d: 0.35) for a repeated measure within factor analysis [24]. To be included in the study, participants needed to: (i) be 45–85 years of age; (ii) present a diagnosis of idiopathic PD (according to the Brain Bank Criteria); (iii) be able to walk independently without devices; (iv) be able to understand verbal commands; (v) be on a stable medication regimen. Volunteers were excluded when presenting: (i) co-morbidities limiting the walking performance; (ii) a Mini-Mental State Examination (M.M.S.E) score lower than 24 [25]; (iii) serious hearing deficits; (iv) walking supports and devices. 

All participants were tested in the ON state of medication, approximately 1-h post first daily dopaminergic medications. Information about participant’s demographic and clinical characteristics are reported in Table 1. 

### 2.2. Additional Clinical Evaluation

The following clinical scales have been administered to each participant to investigate his or her clinical and motor characteristics: (i) Movement Disorder Society-Unified Parkinson’s disease Rating Scale-Part III (MDS-UPDRS-Part III) [26]; (ii) Tinetti Gait and Balance Scale [27]; (iii) Parkinson’s disease quality of life questionnaire (PDQ-39) [28]; (iv) Activities Specific Balance Confidence Scale (ABC) [29]. In order to describe the investigated population, results of the abovementioned scales are reported in Table 2.

### 2.3. Instrumentation

**Figure 1:**
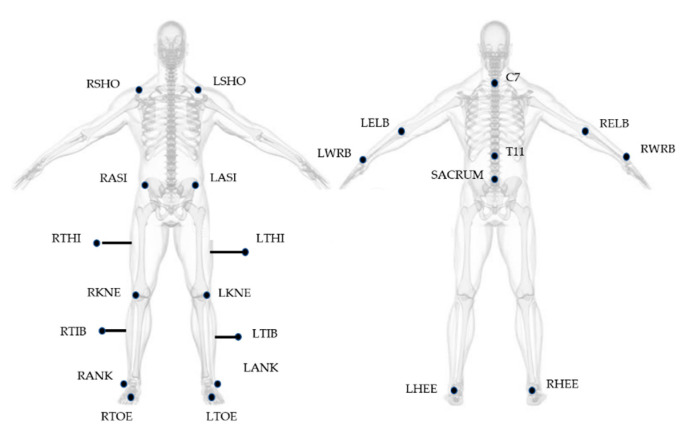
Marker positioning based on Modified Davis Protocol. Letters R and L at the beginning of the acronyms correspond to the body side (right and left). SHO: acromio-clavicular joint; ASI: anterior superior iliac spine; THI: thigh; KNE: lateral epicondyle; TIB: tibiae; ANK: lateral malleoli; TOE: second metatarsal head; C7: 7th cervical vertebrae; T11: 11th thoracic vertebrae; ELB: elbow; WRB: wrist bar pinkie side; HEE: calcaneus.

Gait Analysis was performed at the motion analysis laboratory of IRCCS. Fondazione Santa Lucia in Rome. An optoelectronic system (SMART system, BTS Padova, Italy) and six video cameras (Teli 8320BC), placed along a 10 m walkway with a sampling rate of 50 Hz, were used for the study. The 23 spherical markers (10 mm in diameter) were placed on subject’s body, according to the Modified Davis protocol [30] (Figure 1), with double-sided tape, while thighs and tibial markers were attached approximately 7–10 cm away from the skin on iron sticks, in order to provide more accurate orientation of the segment in three-dimensional space. Laboratory working volume was calibrated prior to each acquisition session by performing an axis and a wand capture.

To improve the accuracy in the positioning of markers, participants’ anthropometric measures were obtained using a taper or a caliper. 

### 2.4. Study Design

**Figure 2:**
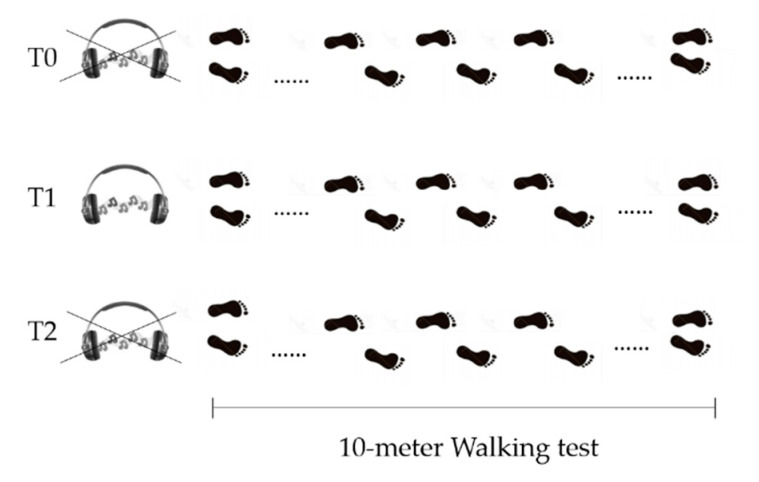
Schematics of study design. T0 = before GR administration. T1= during GR administration. T2 = after GR administration.

In the Gait Analysis laboratory, participants, while barefoot, were asked to perform a linear 10-m walking test, five times, for each of the three following conditions (Figure 2):

1. Before Golden Rhythm (GR) administration (T0): patients were asked to walk at their preferred walking speed. After the five trials, average walking speed was calculated and used to obtain the personalized GR (further information about GR calculation are reported below). The resulting rhythm structure had a duration equal to the gait cycle duration for each patient, with the abovementioned proportion between the two gait phases equal to ϕ.
2. During Golden Rhythm administration (T1): the personalized GR cue was administered to each patient for at least 5 min before the data collection, while they were asked to familiarize with the rhythm while walking. After this time, five walking trials were recorded.
3. After Golden Rhythm administration (T2): the measurement was performed at least 5 min after the stimulus removal. An additional five trials were collected.

At least two strides were recorded for each trial in each condition.

The personalized GR was developed via software by an application reproducing two beats corresponding to the following step events: heel strike (HS) and toe off (TO), for each foot separately. According to the gait cycle, the beat sequence was the following: HS-TO-HS, with a ratio between the temporal distance of HS-TO and that of TO-HS equal to ϕ. The rhythms duration was personalized in accordance with the individual gait speed recorded during the first recording session (T0). 

### 2.5. Data Analysis

The BTS Smart Tracker and BTS Smart Analyzer software were used to analyze the collected data. Gait cycle events (right heel strike, right toe off, left heel strike, and left toe off) were identified by the operator using BTS Smart Analyzer. The measurement area was located in the middle of the 10m walkway to rule out possible acceleration and deceleration effects, enabling the recording from 2 to 4 strides. The BTS software provided spatial and temporal features, which are listed in Table 3. 

### 2.6. The Golden Ratio

The golden ratio (ϕ) is the solution of the problem already reported by Euclid in III century B.C. to cut a given straight line so that the proportion between the shorter part to the longer one is the same as the longer part to the whole. It is an irrational number already found in many physical, biological fractal structures that are self-organized so that the larger-scale structure resembles the subunit structure [31]. 

During walking, a stride is defined as that part of a gait cycle going from a heel strike to the consecutive strike of the same foot, and it is characterized by two main phases: the stance, when the foot is in contact with the ground, and the swing, when the foot moves forwards in the air. These two phases are divided by the toe off, when the foot leaves the ground. Walking is characterized also by the presence of a double support phase, when both feet are in contact with the ground. This phase, absent for example during running, implies that the stance phase should be longer than the swing one. It makes it impossible that the gait cycle is divided by the toe off in two symmetric parts. It has been shown that during comfortable walking of healthy subjects, the toe off divides the stride as the golden section divides the Euclid segment: in a longer part (the stance) that is with the shorter one (the swing) in the same proportion as the entire segment (the stride) is with the longer part (the stance) [21]. For the auto-similar property of this number, it also implies that the same proportion can be find also between swing and double support phase [21].

The following formula (Equation (1)) reports the abovementioned fractal structure of physiological walking and its relationship to the irrational number φ, approximately 1.618, and known as golden ratio [21].

(1) $$\phi =\frac{1+\sqrt{5}}{2}=\frac{swing\;time}{\mathrm{double}\;\mathrm{support}\;\mathrm{time}}=\frac{stance\;time}{swing\;time}=\frac{stride\;time}{stance\;time}$$

Equation (1): Golden Ratio ϕ

Furthermore, in order to show how big the change is relative to the initial value, Delta X, or the change in X, has been calculated: Delta X is mathematically equivalent to X(final) − X(initial), but since we were interested in calculating the percentage change in X, we used the proposed equation (Equation (2))

[X(final) − X(initial)]/X(initial) × 100(2)

Equation (2): Formula of Delta X calculation, where X is one of the investigated variables

Gait parameters, as well as stride-to-stance ratio, were computed for right and left legs, but further referred to as more and less affected sides, based on the distance between the measured stride-to-stance ratio and ϕ (i.e., if right stride-to-stance ratio was 1.31 and the left one was 1.51, the right leg was considered the more affected as it is more far from 1.618). 

### 2.7. Statistical Analysis

The statistical analysis has been performed using the IBM SPSS Statistics v24 software (IBM Corp., Armonk, NY, USA). The normality of the distribution of the variables was verified with the Shapiro–Wilk test. The alpha level for statistical significance was set to 0.05 for all analyses. Since parameters were not normally distributed, the non-parametric equivalent to repeated measures ANOVA was performed: the Friedman Test. For the post-hoc analysis, the Wilcoxon Signed Rank test was used, and Holm–Bonferroni correction was adopted, to counteract the problem of multiple comparisons. Spearman coefficient (Rho) was computed for assessing correlations between stride-to-stance ratio of both more and less affected sides and the other computed variables.

## 3. Results

### 3.1. Comparison of Computed Parameters before, during, and after GR Administration 

**Figure 3:**
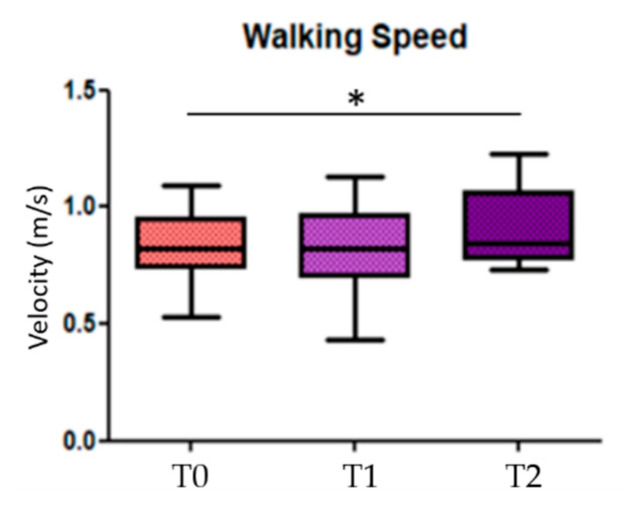
Walking speed. T0 = before Golden Ratio administration; T1 = during Golden Ratio administration; T2 = after Golden Ratio administration; * indicates statistically significant differences.

**Figure 4:**
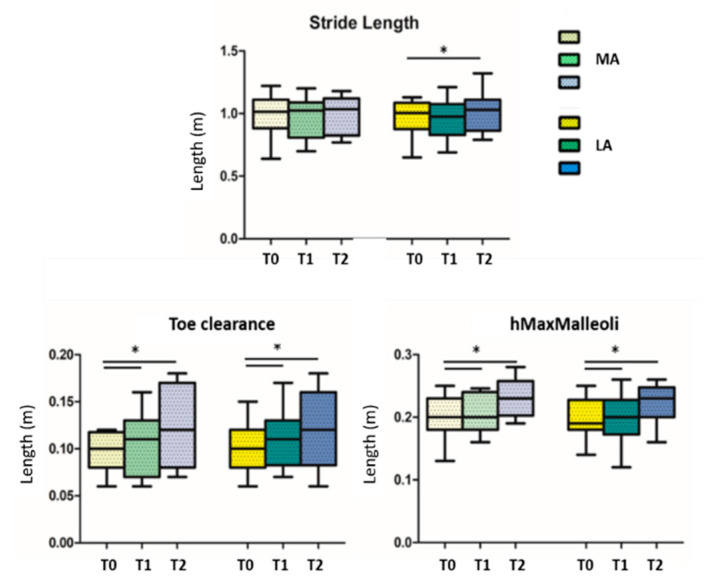
Stride length, toe clearance and maximum height of malleoli are reported; T0 = before GR administration; T1 = during GR administration; T2 = after GR administration; MA = more affected side; LA = less affected side; * indicates statistically significant differences.

When looking for differences among the three conditions (before, during, and after GR), results showed significant statistical differences in the comparison T0 vs. T2 in the walking speed parameter (*p* = 0.014) (Figure 3); the others statistical significant parameters, divided in more and less affected side, are the stride length of the less affected side (*p* = 0.018) in the comparison T0 vs. T2, the *toe clearance* and the *hMaxMalleoli* for both more affected and less affected sides when comparing T0 vs. T1 (*p* = 0.013 and *p* = 0.032, respectively), as well as for T0 vs. T2 (*p* = 0.026 and *p* = 0.033, respectively) (Figure 4). No significant statistical differences have been found for the stride-to-stance ratio. 

### 3.2. Correlations before, during and after Golden Rhythm Administration

Correlations results of T0, T1, and T2 showed that the stride-to-stance ratio of both more and less affected sides well correlated with almost all calculated parameters (Table 4). For the sake of clarity, only parameters with at least one significant correlation with GR were reported.

### 3.3. Correlations of Deltas before, during and after Golden Rhythm Administration

When considering Delta correlations, interesting results have been found when considering patients’ more affected side: in fact, in the Delta correlations T2-T1 and T2-T0, the GR presents correlations only with the stance time (s) and the swing time (%stride) of the more affected side. In the Delta correlation T1-T0, on the other hand, GR shows several correlations with almost all spatio-temporal parameters, in addition to age and disease duration. The full report of correlations is reported in Table 5. For clarity, only parameters with at least one significant correlation with GR were reported.

## 4. Discussion

The aim of the study was to verify if the administration of an ad-hoc auditory stimulus, based on the personalized physiological intrinsic Golden Rhythm, would have an acute effect in the improvement of gait patterns in people with PD. Results show that, despite no differences have been found when comparing the stride-to-stance ratio before, during, and after the ϕ administration, several improvements have been displayed in the other gait parameters. In addition, the GR resulted strongly correlated with almost all spatio-temporal features at T0, T1, and T2, for both more and less affected sides. Interestingly results came from delta correlations: while GR of less affected side showed many correlations in the three delta comparisons, GR of the more affected side was correlated with several parameters in the T1-T0 delta, but only with stance and swing in the T2-T1 and T2-T0 delta correlations. 

Our results confirm that the use of an auditory cue produces immediate effects on several gait variables: as previously reported in the literature, people with PD display particular difficulty with the internal stride length regulation, showing an increase in step cadence to compensate for the reduced step size and gait speed [13]. The administration of the Golden Rhythm possibly helped in increasing sensory and perceptual sensations, which facilitated motor learning [11,18] and consequently produced significant improvements in terms of stride length and walking speed in our patients. Furthermore, the stride-to-stance cue produces improvement in the height of malleoli markers, which significantly changed during and after the GR compared to the T0. Lateral malleoli markers are representative of a clinical feature of PD, shuffling gait. As well known, gait of people with PD appears as if the person is dragging his or her feet while walking, and this characteristic of gait is strictly related to a high risk of falls. Changes shown by PD participants in our study indicates a reduced shuffle gait.

Another important parameter, namely the toe clearance, showed improvements during and after the GR administration compared to T0. This parameter is a representation of limb elevation during gait and a factor likely contributing to the high prevalence of trips in PD [32]. Unanticipated contact with the ground is one preceding event leading to potential postural disturbance: stride-to-stance rhythm seems to promote adequate foot clearance, required to preserve locomotor stability. Overall, the comparison between the three experimental sessions highlighted the presence of clear differences in T1, when the GR-based cue was provided, indicating how the patient adapted his or her gait strategy to the external stimulus. Interestingly, these adaptations were partially maintained in T2, when the GR-based cue was removed, highlighting the acute effects induced by the adopted personalized cue that could represent the potential adaptations possibly induced by the same rhythms when applied along a long-lasting GR-based rehabilitation treatment.

In addition, in order to observe if ϕ was correlated with the other computed parameters, correlation analyses were performed. As first step, correlations at T0, T1, and T2 were taken into account: as hypothesized, the stride-to-stance parameter of both more and less affected sides were correlated with stance and swing parameters, but also with almost all other parameters. Therefore, we decided to perform correlations using deltas (T1-T0, T2-T1, T2-T0, as explained in the methods section), in order to evaluate the presence of possible changes among the evaluations. Interesting results came out: while in the T2-T0 and T2-T1 correlations the GR of the more affected side correlated with stance and swing parameters, in T1-T0 the same GR correlated with almost all considered parameters. This T1-T0 correlations using the deltas interestingly pointed out a very good link with the most common clinical signs, as age and disease duration, and with GR. This pattern was lost in T2, when the auditory cue ceased. Therefore, this aspect further remarked the presence of acute improvements of the GR-based auditory cue, calling for further studies that, considering chronic treatments involving the same auditory stimulus, could prove its potential in inducing long-lasting effects.

Despite the effects on the stride-to-stance ratio were not statistically significant, the slight changes observed in this parameter seemed to be effective in causing a significant difference in other parameters, strongly correlated with the fluidity and stability of gait. 

A larger study enrolling other stages of Hoehn and Yahr may be needed in order to better understand the effect of GR over gait parameters in different phases of PD. Moreover, a new GR may be administered during a standard 10 session or more rehabilitation program, as well as for a longer session. Even electromyographic systems may be used in order to investigate whether GR affects co-contraction as well as extrapyramidal rigidity in PD. In addition, the use of wearable devices would help in moving the acquisition outside the lab environment and, therefore, in a more ecological environment.

### Study Strengths and Limitations

As strength point, this study exhibits motor learning effects that can be visible online, while the patient listens to the GR-based auditory cues, similarly to what was highlighted in the literature using standard metronome-based rhythms. As additional aspect, an after-effect is observed when the auditory cue is removed, with some gait analysis parameters still maintaining their modifications with respect to the initial condition. It is worth noting that these modifications can be already obtained in only one single session of auditory cue administration. 

On the other hand, the stride-to-stance ratio did not show up any significant changes, as it happens for many other gait parameters. Due to the nature of this parameter and its small achievable changes, longer GR-administration times would be necessary to observe significant differences in this parameter.

## 5. Conclusions

The instrumental approach proposed in this work supports the following finding: the Golden Rhythm seems to be an effective external cue able to compensate for the defective internal rhythm of the basal ganglia in people with PD. Conversely to classic auditory cues (i.e., metronome), the stride-to-stance rhythm is a personalized stimulus based on individual’s intrinsic fractal structure of gait, therefore it describes a more complex structure of gait than the only symmetry. A rehabilitation pathway based on the repetition of this stimulation session could be even more effective for improving gait parameters of patients with Parkinson’s Disease than traditional cues.

## Figures and Tables

**Table 1:** Mean and standard deviation values are displayed at the top of the table. M = males; F = females; PD Duration: years of Parkinson’s disease duration; LD Duration: years of Levodopa treatment duration.

	Age [yrs] 70.9 (8.4)	Gender 16 M,4 F	Height [cm] 167.3 (8.8)	Body Mass [kg] 69.4 (10.9)	PD Duration [yrs] 10.0 (6.2)	LD Duration [yrs] 9.4 (5.6)
1	83	M	176	73	5	5
2	63	F	167	58	10	9
3	72	M	168	64	13	12
4	78	F	150	49	8	8
5	59	M	158	60	20	20
6	67	M	165	61	25	20
7	63	M	171	67	15	15
8	77	M	169	67	9	9
9	53	F	152	51	7	7
10	69	M	170	63	10	8
11	64	M	181	79	11	10
12	70	M	175	91	10	10
13	83	M	162	65	12	11
14	71	F	155	76	4	4
15	68	M	164	77	7	7
16	76	M	180	70	1	1
17	79	M	165	75	7	6
18	85	M	173	77	6	6
19	65	M	178	78	1	1
20	67	M	168	87	20	20

**Table 2:** Obtained clinical scale scores. Mean and standard deviation values are reported. For all clinical scales, the minimum value of the range indicates the worst performance.

Clinical Scale	Range	Score
MDS-UPDRS (Part III)	0–132	22.5 (6.7)
MMSE	0–30	22.5 (2.2)
Tinetti Gait and Balance	0–28	22.4 (4.7)
PDQ-39	0–100	34.5 (19)
ABC	0–100	39.0 (16.0)

**Table 3:** Calculated features and corresponding measurement units.

Features	Measure	Features	Measure
Stride time	s	Walking Speed	m/s
Stance time	s	Cadence	Steps/min
Stance Percentage	%stride	Width of the step	m
Stride length	m	Golden Ratio (ϕ)	Stride/stance
Swing time	s	Lateral Malleoli displacement (*hMaxMalleoli*)	m
Swing Percentage	%stride	Arm Swing (*hMaxWrist*)	m
Double support time	s	Toe Clearance second hallux (Toe Clearance)	m
Double support Percentage	%stride		

**Table 4:** Rho Spearman coefficient is reported in the table. T0 = before GR administration; T1 = during GR administration; T2 = after GR administration; MA = more affected side; LA = less affected side; * = Correlation is significant at the 0.05 level; ** = Correlation is significant at the 0.01 level.

		T0		T1		T2	
		Golden Ratio (MA)	Golden Ratio (LA)	Golden Ratio (MA)	Golden Ratio (LA)	Golden Ratio (MA)	Golden Ratio (LA)
Age		−0.529 *	−0.402	−0.529 *	−0.402	−0.404	−0.406
MDS-UPDRS		−0.213	−0.297	−0.213	−0.297	−0.373	−0.474 *
Disease duration		0.436	0.449 *	0.436	0.449 *	−0.006	0.207
Stride time	MA	−0.116	−0.332	−0.116	−0.332	−0.348	−0.233
	LA	−0.209	−0.305	−0.209	−0.305	−0.498 *	−0.214
Stance Percentage	MA	−0.825 **	−0.607 *	−0.825 **	−0.607 **	−0.998 **	−0.277
	LA	0.777 **	−0.868 **	−0.771 **	−0.868 **	−0.310	−0.995 **
Swing Percentage	MA	0.825 **	0.607 *	0.825 **	0.607 *	0.998 **	0.277
	LA	0.777 **	0.853 **	0.777 **	0.853 **	0.308	0.995 **
Double Support Time	MA	−0.663 **	−0.877 **	−0.663 **	−0.877 **	−0.487 *	−0.794 **
	LA	−0.540 *	−0.421	−0.540 *	−0.421	−0.720 **	−0.600 **
Step width		0.455 *	0.188	0.455 *	0.188	0.606 **	0.358
Stride length	MA	0.361	0.319	0.361	0.310	0.469 *	0.191
	LA	0.411	0.349	0.411	0.349	0.412	0.132
ϕ	MA	1	0.675 **	0.675 **	1	1	0.305
	LA	0.675 **	1	1	0.675 **	0.191	1
*hMaxWrist*	MA	−0.031	−0.468 *	−0.031	**−0.468**	0.229	−0.111
	LA	−0.027	−0.280	−0.026	−0.012	−0.053	0.072
Walking speed		0.324	0.364	0.324	0.364	0.564 **	0.275

**Table 5:** Rho Spearman coefficient of Delta correlations is reported in the table. T0 = before Golden Rhythm administration; T1 = during Golden Rhythm administration; T2 = after Golden Rhythm administration; MA = more affected side; LA = less affected side; * = Correlation is significant at the 0.05 level; ** = Correlation is significant at the 0.01 level.

		T1-T0		T2-T1		T2-T0	
		Golden Ratio (MA)	Golden Ratio (LA)	Golden Ratio (MA)	Golden Ratio (LA)	Golden Ratio (MA)	Golden Ratio (LA)
Age		0.467 *	−0.048	0.211	0.119	0.081	0.211
Disease duration		−0.568 **	−0.529 **	−0.311	−0.117	−0.112	−0.231
Stride time	MA	−0.460 *	−0.295	−0.039	−0.229	−0.039	−0.229
	LA	−0.542 *	−0.283	−0.229	−0.185	−0.229	−0.185
Stance Percentage	MA	−0.941 **	−0.439	−0.630 **	−0.278	−0.630 **	−0.278
	LA	−0.794 **	−0.791 **	−0.353	−0.853 **	−0.353	−0.853 **
Swing Percentage	MA	0.920 **	0.400	0.738 **	0.180	0.737 **	0.180
	LA	0.776 **	0.800 **	0.229	0.869 **	0.259	0.869 **
Double Support Time	MA	−0.830 **	−0.544 *	−0.340	−0.797 **	−0.340	−0.797 **
	LA	−0.645 *	−0.250	−0.317	−0.226	−0.317	−0.226
Stride length	MA	0.502 *	0.373	−0.197	0.057	−0.197	0.057
	LA	−0.003	0.247	−0.382	−0.113	−0.382	−0.113
ϕ	MA	1	0.582 **	1	0.266	1	0.266
	LA	0.582 **	1	0.266	1	0.266	1
Toe Clearance	MA	−0.496 *	−0.293	−0.354	−0.038	−0.354	−0.038
	LA	−0.185	0.167	−0.221	0.111	−0.221	−0.121
Walking speed		0.364	0.307	0.081	0.621 **	0.081	0.523 **

## Data Availability

Not applicable.
